# A rice tonoplastic calcium exchanger, OsCCX2 mediates Ca^2+^/cation transport in yeast

**DOI:** 10.1038/srep17117

**Published:** 2015-11-26

**Authors:** Akhilesh K. Yadav, Alka Shankar, Saroj K. Jha, Poonam Kanwar, Amita Pandey, Girdhar K. Pandey

**Affiliations:** 1 Department of Plant Molecular Biology, University of Delhi South Campus, Benito Juarez Road, Dhaula Kuan, New Delhi-110021, India

## Abstract

In plant cell, cations gradient in cellular compartments is maintained by synergistic action of various exchangers, pumps and channels. The *Arabidopsis* exchanger family members (AtCCX3 and AtCCX5) were previously studied and belong to CaCA (calcium cation exchangers) superfamily while none of the rice CCXs has been functionally characterized for their cation transport activities till date. Rice genome encode four CCXs and only *OsCCX2* transcript showed differential expression under abiotic stresses and Ca^2+^ starvation conditions. The OsCCX2 localized to tonoplast and suppresses the Ca^2+^ sensitivity of K667 (low affinity Ca^2+^ uptake deficient) yeast mutant under excess CaCl_2_ conditions. In contrast to AtCCXs, OsCCX2 expressing K667 yeast cells show tolerance towards excess Na^+^, Li^+^, Fe^2+^, Zn^2+^ and Co^2+^ and suggest its ability to transport both mono as well as divalent cations in yeast. Additionally, in contrast to previously characterized AtCCXs, OsCCX2 is unable to complement yeast *trk1trk2* double mutant suggesting inability to transport K^+^ in yeast system. These finding suggest that OsCCX2 having distinct metal transport properties than previously characterized plant CCXs. OsCCX2 can be used as potential candidate for enhancing the abiotic stress tolerance in plants as well as for phytoremediation of heavy metal polluted soil.

Plants require various essential cations for numerous cellular metabolic activities, growth and development. Presence of excess essential ions in the cell leads to ion toxicity while concentration below optimal level leads to ion deficiency symptoms. Therefore, plants have employed wide range of mechanism to uptake mineral nutrients from soil by various transporters presents on the plasma membrane of epidermal root cells. Subsequently minerals from the roots are translocated to different plant organs and tissues for plant growth and development^1^.

Calcium (Ca^2+^) is an essential mineral nutrient as well as a pivotal second messenger in plant cells^2^,^3^. Upon perception of various stimuli, transient increase in [Ca^2+^]_cyt_ leads to activation of various signal transduction pathways in the plant cells, which regulates the various cellular mechanisms such as opening and closing of stomatal aperture^4^, self-incompatibility during fertilization^5^, development of root hairs and pollen tube growth and guidance^6^, light and circadian signaling^7^,^8^,^9^, hyperosmotic and oxidative stresses^10^, different abiotic stress responses as well as interaction with pathogenic and symbiotic microorganisms^11^,^12^. Therefore, study of calcium transport mediated by different family of calcium transporters in the cell is an important aspect in biological processes.

In soil, primarily Ca^2+^ absorbs in root by synergistic action of Ca^2+^-permeable transporters and translocated to shoot either by apoplastic or symplastic transport^10^,^13^. In the resting cells, submicromolar [Ca^2+^]_cyt_ is required to regulate various signaling pathways^14^,^15^,^16^. In contrast to resting stage of cells, when plants are exposed to various external stimuli like biotic, abiotic, nutrient deficiency, or developmental cues, [Ca^2+^]_cyt_ level is increased to several hundred folds which generates ‘calcium signature’^17^. This ‘calcium signature’ leads to activation of various signaling pathways. This [Ca^2+^]_cyt_ burst is normalized by synergistic action of low capacity, high affinity (Km = 1–10 μM) calcium ATPases and high capacity, low affinity (Km = 10–15 μM) calcium exchangers (CAXs)^13^,^17^,^18^,^19^. Therefore, Ca^2+^-ATPases and CAXs maintain optimum [Ca^2+^]_cyt_ either by apoplastic export or by sequestering excess Ca^2+^ to the lumen of vacuole against electrochemical gradient in the activated plant cells^15^,^20^,^21^.

The cation/Ca^2+^ (CaCA) superfamily exchangers are calcium transporters, which play an essential role in calcium signaling pathways in many organisms^22^. The CaCA superfamily members have been identified widely in bacteria, archaea, fungi, animals and plants. CaCA superfamily is classified into five families, as YRBG (named after yrbG of *E. coli,* a putative Na^+^/Ca^2+^ exchanger), NCX (K^+^-independent Na^+^/Ca^2+^ exchangers), NCKX (K^+^-dependent Na^+^/Ca^2+^ exchangers), CAX (H^+^/cation exchangers) and CCX (cation/Ca^2+^ exchangers)^22^,^23^. The CaCA exchangers transport Ca^2+^ across various membranes against its electrochemical gradient by utilizing the downhill gradient of other cation species such as H^+^, K^+^ or Na^+^ 22. Structurally, CaCA superfamily is defined by the presence of two highly conserved α-repeat hydrophobic domains, important for ion selectivity/binding/transport are separated by a central hydrophilic loop^21^,^22^,^24^,^25^,^26^. Earlier studies also suggest the evolutionary significance of various plant CAXs and CCXs for their structural and functional similarity and divergence^23^,^27^.

Previously, *Arabidopsis* calcium/cation exchangers (CCXs) were identified as CAX homologs but recently CAX7 to CAX11 were reclassified as CCX1 to CCX5 due to higher homology to mammalian NCKX (K^+^-dependent Na^+^/Ca^2+^ antiporters)^28^. Several plant CAXs are vacuole localized cation/H^+^ antiporters that mediates H^+^-coupled antiport of Ca^2+^ and other metal ions and recognised as high capacity, low-affinity transporter resulting in the accumulation of these cations in vacuoles^21^,^29^,^30^,^31^. Till date, only two plant CCXs (AtCCX3 and AtCCX5 of *Arabidopsis*) have been studied. AtCCX3 is an endomembrane localized H^+^ dependent K^+^ transporter, which suppresses yeast mutants defective in Na^+^, K^+^, and Mn^2+^ transport and therefore, it is a K^+^ transporter with apparent Na^+^ and Mn^2+^ transport properties^32^. AtCCX5 has been characterized in yeast system where AtCCX5::GFP fusion protein localize to plasma membrane and nuclear periphery, involved in high-affinity K^+^ uptake and Na^+^ transport^33^. Overall, *Arabidopsis* CCX3 and CCX5 belong to the same family having a few similarities and distinction in cation transport properties^32^,^33^. The rice genome encode four putative CCXs (OsCCX1-OsCCX4)^23^,^27^,^34^. Till date none of the rice CCXs is studied and it would be interesting to study CCX from crop plant to compare their activities with already characterized *Arabidopsis* CAXs and CCXs.

Studies in yeast support the role for *Arabidopsis* CCXs in cation homeostasis while possible functions of rice CCXs are not characterized yet. To understand the functional role of rice CCXs, we have performed detailed expressional analysis of all four rice CCXs in abiotic stresses and calcium deficiency and found that only *OsCCX2* showed differential expression under above mentioned conditions. We observed the subcellular localization of OsCCX2 to vacuolar membrane in plant epidermal cells. For functional characterization of OsCCX2, we did complementation analysis of OsCCX2 in heterologous yeast system to ascertain its role in transporting Ca^2+^, Na^+^, Li^+^ and distinct heavy metal ions (Fe^2+^, Zn^2+^ and Co^2+^) in appropriate yeast mutants.

## Results

### OsCCX2 transcript is differentially expressed under abiotic stresses

**Figure 1:**
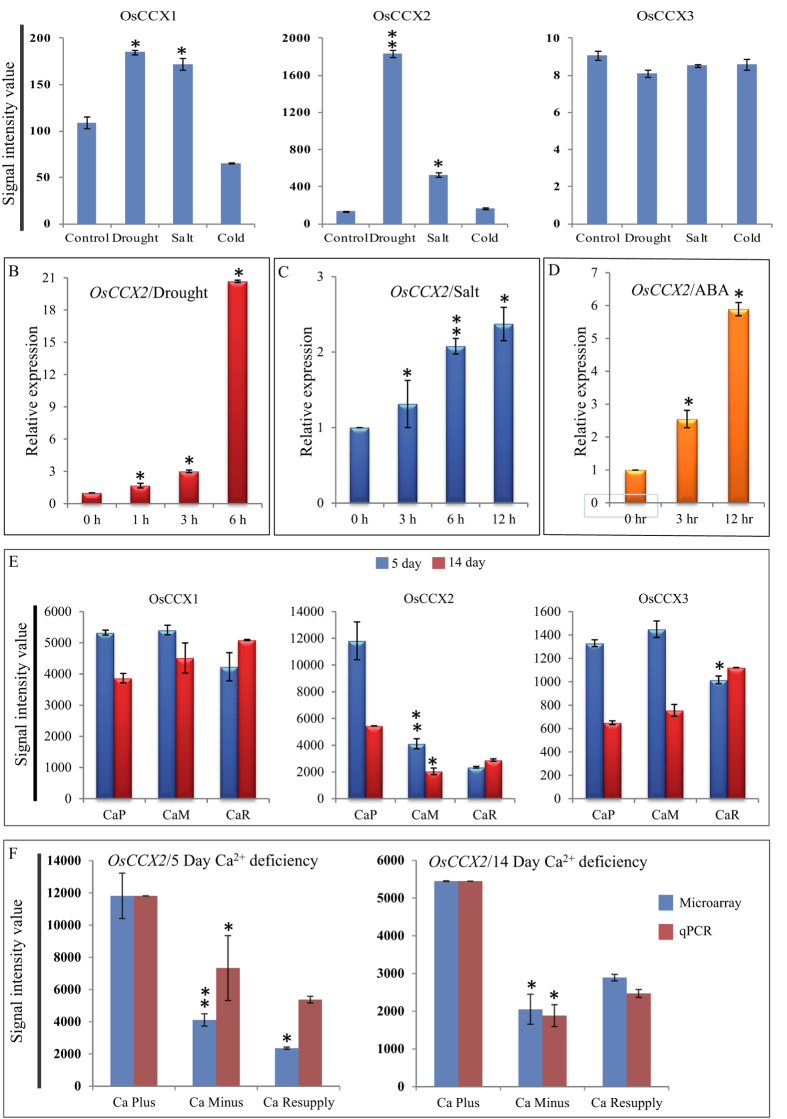
Expression profile of OsCCX2 under abiotic stresses and Ca^2+^ deficiency. (**A**) Microarray expression of *OsCCX1*, *OsCCX2* and *OsCCX3* under drought, salt and cold conditions. (**B**) The quantitative PCR validation of *OsCCX2* expression under drought (**C**), salt and (**D**) exogenous ABA treatments. X-axis denotes different time points and Y-axis depicts the relative expression value in terms of fold change. (**E**) The microarray expression profile of *OsCCX1*, *OsCCX2* and *OsCCX3* under Ca^2+^ deficiency. Among *OsCCXs*, *OsCCX2* shows distinct and differential expression after 5 and 14 days of Ca^2+^ deficiency and 6 hrs of Ca^2+^ resupply. X-axis represents different nutritional treatments and Y-axis represents signal intensity value. (**F**) The microarray expression profile of *OsCCX2* was validated under similar conditions using qPCR. Three biological replicates were used for both microarray and qPCR analysis and their mean ± SD values are plotted. X-axis represents different nutritional treatments and Y-axis represents relative expression values obtained after normalizing the data against maximum microarray expression value. Blue bars represent the expression from microarrays, while red bars represent the qPCR values. Differences between mean values of treatments and controls were compared using Student’s t -tests (^*^P≤0.05, ^**^P≤0.01).

The expression profile of a gene provides a clue about its functional relevance and paves way for its further characterization. Four *CCX* genes namely *OsCCX1, OsCCX2, OsCCX3* and *OsCCX4* were identified in rice genome. The probesets of *OsCCX1* to *OsCCX3* were available while probesets for *OsCCX4* were not labelled on microarray chip (Agilent and Affimetrix). To determine the transcript expression profile of *OsCCX1*-*3* under abiotic stresses (drought, salt and cold), publically available rice microarray database, Rice DB:Oryza information portal (http://ricedb.plantenergy.uwa.edu.au/) was used. The *OsCCX2* transcript was differentially upregulated under drought and salt stresses while no significant change was observed under cold stress (Fig. 1A). The expression profile of two other *CCXs*, *OsCCX1* and *OsCCX3* was not significantly changed under drought, salt and cold stresses (Fig. 1A). Expression data of 3 hrs exogenous application of ABA to rice seedlings^35^ showed that transcript of *OsCCX2* was highly upregulated while *OsCCX3* transcript was downregulated (Fig. S1a). The microarray expression profiles of *OsCCX2* in drought, salt and ABA treatment were also validated by qPCR and transcript was found to be upregulated upon longer exposure to these stresses (Fig. 1B–D). Seedlings treated with longer exposure to drought depict increasing transcript abundance and after 6 hrs of drought stress, *OsCCX2* transcript shows more than twenty fold increase in transcript abundance (Fig. 1B). Likewise, *OsCCX2* transcript showed gradual transcripts abundance under longer exposure to salt or exogenous ABA application (Fig. 1C,D). The results were statistically supported with significant p-value. ABA and drought responsive transcript abundance is further strengthened by presence of various ABA responsive and dehydration responsive elements in 1 kb upstream promoter region of *OsCCX2* (Fig. S1b).

### OsCCX2 transcript is down-regulated under Ca^2+^ deficiency

To understand the calcium dependent function of rice *OsCCXs*, microarray data^36^ was analysed to determine the expression profile of these genes under Ca^2+^ deficient conditions. Under Ca^2+^ deficiency conditions of 5 and 14 days, the transcript levels of *OsCCX1* and *OsCCX3* remained unaffected, whereas *OsCCX2* showed significant down regulation. However, *OsCCX2* showed upregulation after 6 hrs of Ca^2+^ resupply to prolonged Ca^2+^ deficiency (14 days) treated seedling, indicating role of *OsCCX2* in calcium signaling and transport (Fig. 1E). The microarray expression profile of *OsCCX2* was validated by qPCR showing similar expression profile under Ca^2+^ deficiency and 6 hrs of Ca^2+^ resupply conditions (Fig. 1F). Among all rice *CCXs*, only *OsCCX2*, showed a distinct and differential expression pattern under Ca^2+^ deficiency. The results were statistically supported with significant p-value. This down regulation in transcript abundance of *OsCCX2* is indirectly supported by presence of various calcium responsive and CaM binding elements such as ABRERATCAL and CGCGBOXAT^37^,^38^ in 1 kb upstream promoter region of *OsCCX2* (Fig. S1b). The presence of three ABRERATCAL elements and sixteen CGCGBOXAT boxes in the promoter region of *OsCCX2* signify its role in calcium responsive pathways. Along with Ca^2+^ binding and ABA responsive elements, we also analysed the presence of nutrition responsive *cis*-acting elements and four sulphur responsive element, SURECOREATSULTR11, were found at 1 kb upstream of *OsCCX2* gene but other nutrition responsive elements were absent. We have also analysed the expression data of rice CAXs and CCXs under different nutrient deficient conditions. The *OsCCX2* was up-regulated under low phosphate (P) conditions (72 h, 24 h and 6 h) while down regulated in shoot under P deficiency. OsCCX2 was also down regulated under potassium (K^+^) deficiency. The expression of other rice CCXs were not much affected in major nutrient deficient conditions. The expression of rice *CAX1a, CAX1b* and CAX1c were altered in low P and Fe (iron) conditions while transcripts of other rice CAXs were not much affected under different nutrient deficient conditions (Fig. S2).

### OsCCX2 localized to tonoplast in *N. benthamiana* epidermal cells

**Figure 2:**
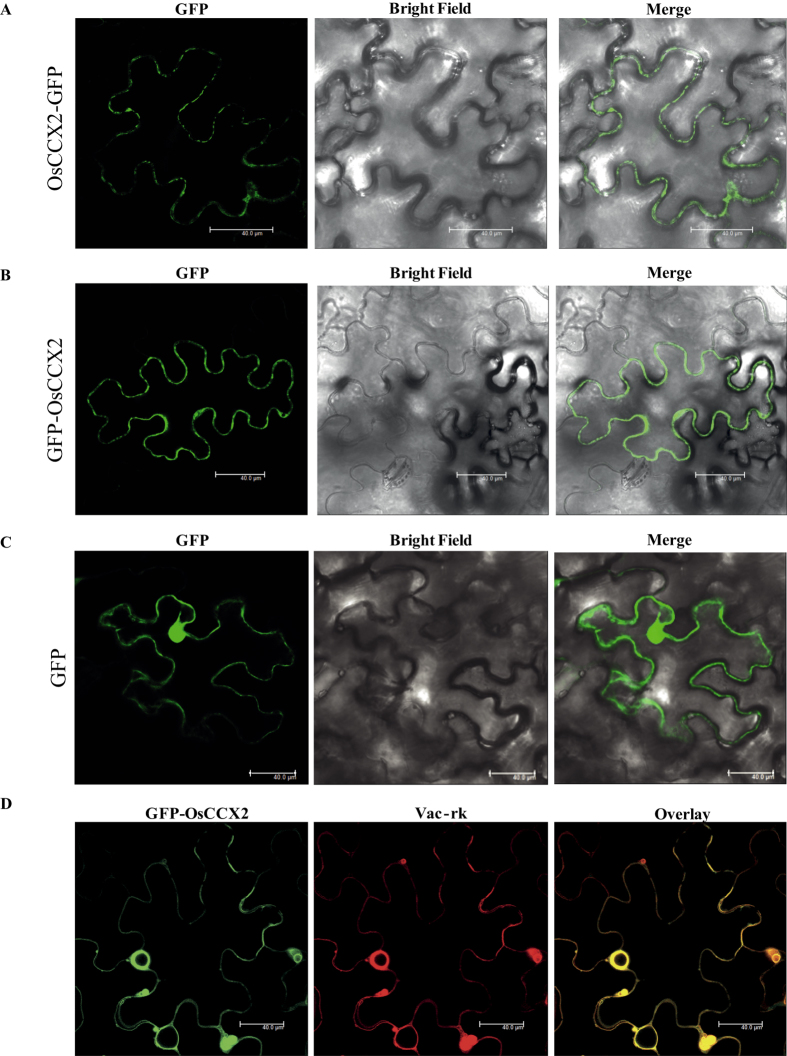
Subcellular localization of OsCCX2 proteins in *Nicotiana benthamiana* epidermal cells. (**A**) OsCCX2 fused with N-terminal of GFP appears as circular pre-vesicles inside the lumen of the cell and seems to be localized to vacuolar membrane. (**B**) The OsCCX2 fused at the C-terminal of GFP also showed tonoplast localization. (**C**) Cells transformed with CaMV 35S -GFP were used as a control. Fluorescence was detected under a confocal laser-scanning microscope (wavelength: 488 nm). (**D**) The GFP-OsCCX2 co-localized completely with globular vesicles as shown by tonoplast markers (vac-rk). GFP fusions to OsCCX2 proteins are shown in green, mCherry vacuole markers are shown in red and overlay of two-mentioned proteins in dark field view. Scale bar = 40 μm.

To investigate the function of a gene at protein level, it is important to know about its possible site of residency in the cell. Especially, in plant cells calcium is stored and released from different intra- and extra- cellular storage compartments like vacuole, endoplasmic reticulum (ER), mitochondria, chloroplasts and cell wall^2^,^17^,^39^. Therefore, understanding of the sub-cellular localization of various types of Ca^2+^ transporters becomes more relevant. To determine the sub-cellular localization, OsCCX2::GFP and GFP::OsCCX2 fusion proteins were generated. The 35 S CaMV promoter driven GFP fluorescence of empty vector control (pGPTVII.GFP.Kan and pSITE2CA) was localized throughout the cytosol and the nucleus. GFP fluorescence of either OsCCX2::GFP or GFP::OsCCX2 was shown at vacuolar membrane in transient *Agrobacterium* mediated transformation of *N. benthamiana* epidermal peel cells (Fig. 2). To confirm the vacuolar localization of OsCCX2, we did co-localization with standard vacuolar marker, vac-rk (CD3-975, ABRC). Upon transient *Agrobacterium* mediated cotransformation of *N. benthamiana* epidermal peel cells with GFP::OsCCX2 and vacuolar marker, vac-rk, the GFP fluorescence of GFP::OsCCX2 is completely merged with RFP fluorescence of tonoplast marker vac-rk, confirming the localization of OsCCX2 to the vacuolar membrane and probable site of action for this protein.

### OsCCX2 has characteristic Ca^2+^/cation transport signature elements

Calcium exchanger proteins of rice comprised of six Ca^2+^/H^+^ exchangers (CAXs), four cation/Ca^2+^ exchangers, two EF-CAX, two MHX and single OsNCKX1^23^,^27^. The *OsCCX2* (LOC_Os03g45370) open reading frame contains 1,728 nucleotides, translating into a putative 60 kDa protein (576 amino acids) and its domain analysis reveals that it is a putative Na^+^/Ca^2+^ exchanger protein. Domain structure analysis of OsCCX2 reveals that it has typical 12 transmembrane domains, two Na^+^/Ca^2+^ exchanger domains and characteristic α-1 and α-2 motifs, which are considered necessary and sufficient for cation exchange activity. The presence of unique signature motifs GNG(A/S)PD in α-1 and (G/S)(N/D)SxGD motif in α-2 repeat regions^23^,^28^ also provide indirect evidence regarding Ca^2+^ ion selectivity, binding, transport of OsCCX2 protein (Fig. S3).

It shows more than 60% protein sequence homology with cation/Ca^2+^ exchanger of monocot species (*Brachypodium*, *Sorghum* and *Zea mays*) and 41% protein sequence homology with *Arabidopsis* CCX1. In phylogenetic analysis, OsCCX2 groups with *Arabidopsis* and rice CCXs and its closest *Arabidopsis* orthologs are AtCCX1 and AtCCX2^27^. A high degree of sequence homology among *Arabidopsis* and rice CCXs suggests possible involvement of OsCCX2 in similar calcium signaling pathways and prompted us to perform in-depth functional characterization to understand the cation/Ca^2+^ exchange properties of OsCCX2.

### OsCCX2 suppress Ca^2+^ sensitivity of K667 yeast mutant

Previously, yeast heterologous expression system approach has been used successfully to decipher the functional role of plant exchanger proteins^31^,^32^,^33^,^40^. We adopted the same approach to determine the role of OsCCX2 in transport of Ca^2+^ and other cations. K667, a low affinity Ca^2+^ uptake deficient triple *S. cerevisiae* mutant strain, lacking vacuolar ATPase, PMC1; vacuolar exchanger, VCX1; and cytosolic regulatory CNB1 subunit^41^. The growth of K667 mutant is compromised in media having excess Ca^2+^ concentration and is due to defective vacuolar Ca^2+^ transport^41^.

**Figure 3:**
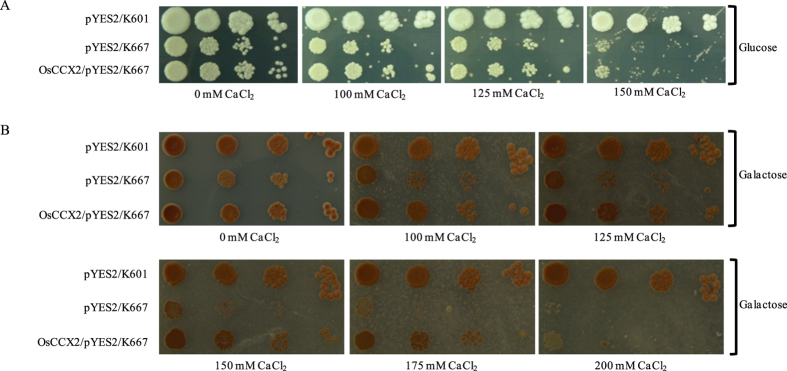
Suppression of Ca^2+^ sensitivity of the K667 yeast mutant by OsCCX2. The yeast K667 triple mutant (∆*pmc1*∆*vcx1*∆*cnb1*) is unable to grow in the presence of higher CaCl_2_ because low affinity Ca^2+^ transport is compromised in the mutant. The galactose inducible yeast expression vector, pYES2, was used to complement K667 with OsCCX2. The growth of pYES2/K667 (empty vector control) was compromised at higher CaCl_2_ concentration either in presence of glucose or galactose in the medium. Yeast strain transformed with either vector or OsCCX2 were grown in SC-Ura media overnight at 30 °C and diluted to 0.5 OD_600_ followed by 10 times serial dilutions of 0.5 OD_600_ were dotted on SC-URA + Gal with different CaCl_2_ concentrations. The plates were incubated at 30 °C for 3 days. A K667 yeast mutant expressing OsCCX2 could grow in presence of excess CaCl_2_ while growth of empty vector expressed mutant was severely compromised.

Moreover, it was also reported that *Arabidopsis* CCX5 and CCX3 were unable to suppress the high Ca^2+^ sensitivity of yeast mutant K667, where the later was characterized as endomembrane localized H^+^ driven K^+^ transporter^32^,^33^. OsCCX2 has similar topology and domain organization like *Arabidopsis* CCXs but OsCCX2 shares only 41% sequence homology with *Arabidopsis* CCX1 and lesser sequence similarity with previously characterized CCX3 and CCX5, indicating that OsCCX2 might have distinct transport activity. Most of the *Arabidopsis* and rice CAXs have Ca^2+^ exchange activity and are able to complement the Ca^2+^ sensitive phenotype of K667 mutant. Till date, plant’s CCXs have not been reported to complements yeast K667 mutant strain. To test whether rice OsCCX2 has the ability to suppress the Ca^2+^ sensitivity of K667, the complete ORF of OsCCX2 was cloned into galactose inducible pYES2 yeast expression vector and expressed in K667 mutant cells along with empty vector as a control. The K667 mutant cells expressing empty vector were unable to grow under excess CaCl_2_ either in presence of glucose or galactose (Fig. 3A,B). The calcium sensitivity of K667 was suppressed by OsCCX2 in presence of galactose but not in glucose; confirming the inducibility and expression of OsCCX2 protein under higher CaCl_2_ concentrations (Fig. 3A,B). It was found that K667 expressing OsCCX2 yeast cells could grow better while growth of empty vector expressing K667 cells was highly compromised at higher CaCl_2_ (up to 150 mM) (Fig. 3B).

Moreover, multiple sequence alignment of *Arabidopsis* CCXs with CAXs revealed that CCXs do not have N-terminal autoinhibitory domain in contrast to CAXs^32^. The full length ORF of OsCCX2 was able to complement K667 mutant, which further indicate that it do not have a N-terminal autoinhibitory domain while showing Ca^2+^ exchange properties like other CAXs.

### OsCCX2 suppress Na^+^ and Li^+^ sensitivity of K667 yeast mutant

**Figure 4:**
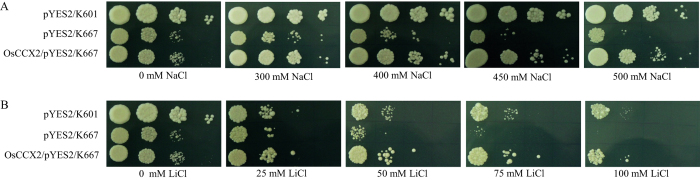
Suppression of Na^+^ and Li^+^ sensitivity of K667 yeast mutant by OsCCX2. Yeast strains having empty vectors and OsCCX2 were grown in SC-Ura overnight at 30 °C and diluted to 0.5 OD_600_ followed by 10 times serial dilutions of 0.5 OD_600_ were dotted on SC-URA+Gal containing different concentrations of NaCl and LiCl. The plates were incubated at 30^0^ C for 3 days. OsCCX2 was able to provide tolerance to K667 yeast mutant under excess NaCl and LiCl conditions.

*Arabidopsis* CAXs and CCXs have broad substrate specificity and can transport a variety of monovalent (Na^+^, K^+^, Li^+^) as well as divalent (Ca^2+^, Mn^2+^, Zn^2+^, Cd^2+^, Co^2+^) cations. Although some CAXs such as AtCAX1 and *Saccharomyces cerevisiae* VCX1 (ScVCX1) are specific for Ca^2^+ 27,29,32,41,42,43 transport, the mammalian CCX family member NCKX6, has been demonstrated to mediate Na^+^/Ca^2+^ and Li^+^/Ca^2+^ exchange^44^. The growth of K667 mutant is also compromised at higher concentration of NaCl^42^. In order to determine the Na^+^ transport properties of OsCCX2, we have performed the complementation assay in K667 yeast mutant at higher concentration of exogenous NaCl. In contrast to empty vector control, OsCCX2 expressing mutant cells grew better on media supplemented with high concentration of NaCl (200–500 mM) (Fig. 4A). This reveal that OsCCX2 efficiently suppress the Na^+^ hypersensitivity of K667 in a manner similar to the suppression of Ca^2+^ sensitive phenotype of this mutant (Fig. 4A). K667 yeast mutant shows higher growth sensitivity to excess LiCl than NaCl under increasing concentration of LiCl from 25 mM to 100 mM (Fig. 4B). Empty vector expressing K667 mutant cells were not able to grow at 100 mM LiCl while growth of OsCCX2 expressing yeast mutant is comparable to wild type strain.

### OsCCX2 provide metal tolerance to K667 yeast mutant

On the basis of phylogenetic analysis of CAXs, type IB CAXs (CAX2, CAX5 and CAX6) are involved in transporting other metal ions with broader substrate specificity for divalent cations, such as Ca^2+^, Mn^2+^, Zn^2+^, Co^2+^ and Cd^2^+^20^,^29^,^31^,^45^,^46^,^47^. Whereas, in the case of CCXs, only AtCCX3 and AtCCX5 show broad range of substrate specificity from monovalent K^+^ and Na^+^ to divalent cations such as Mn^2+^ and Zn^2+ ^32,33.

**Figure 5:**
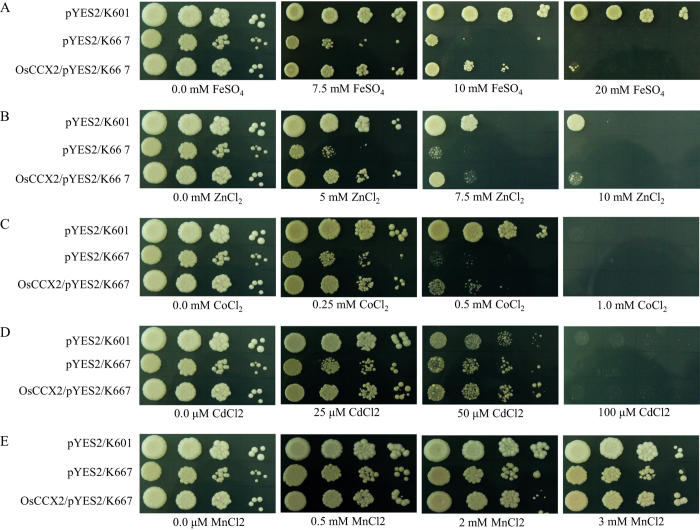
The yeast mutant K667 complemented by OsCCX2 provide tolerance against excess Fe^2+^, Zn^2+^ and Co^2+^. Yeast strains transformed with vectors and OsCCX2 were grown in SC-Ura medium for overnight at 30 °C and diluted to 0.5 OD_600_ and 10 times serial dilutions of 0.5 OD_600_ were dotted on SC-URA+Gal plates containing different concentration of FeSO_4_, ZnCl_2_, CoCl_2_, CdCl_2_ and MnCl_2_. The plates were incubated at 30 °C for 3 days. OsCCX2 was able to suppress the Fe^2+^, Zn^2+^ and Co^2+^ sensitivity of K667 yeast mutant and hence provided tolerance towards these metals ions. OsCCX2 does not provide tolerance against excess CdCl_2_ and MnCl_2_.

In order to understand transport properties of various metal ions (Fe^2+^, Zn^2+^, Co^2+^ Mn^2+^ and Cd^2+^) mediated by OsCCX2, the sensitivity and tolerance of OsCCX2 expressing K667 cells were examined under excess concentrations of above mentioned heavy metals. We found that OsCCX2 expressing K667 yeast cells provide tolerance specifically against excess FeSO_4_, ZnCl_2_, and CoCl_2_ than empty vector control (Fig. 5A–C). The sensitivity of OsCCX2 expressing K667 yeast cells was distinct and varied for different heavy metals (Fig. 5A–E). Like the OsCCX2 expressing K667 cells could grow in 20 mM FeSO_4_ while empty vector control did not show any growth at this concentration (Fig. 5A). OsCCX2 also provides tolerance against 10 mM ZnCl_2_ (Fig. 5B). Similarly, empty vector expressing K667 cells showed impaired growth while OsCCX2 provides tolerance to yeast mutant upto 0.5 mM CoCl_2_ (Fig. 5C). In contrary, OsCCX2 could not provide tolerance to K667 cells under mentioned concentration of Mn^2+^ and Cd^2+^ (Fig. 5D,E).

### OsCCX2 suppress metal ion sensitivity of *mid1* mutant

**Figure 6:**
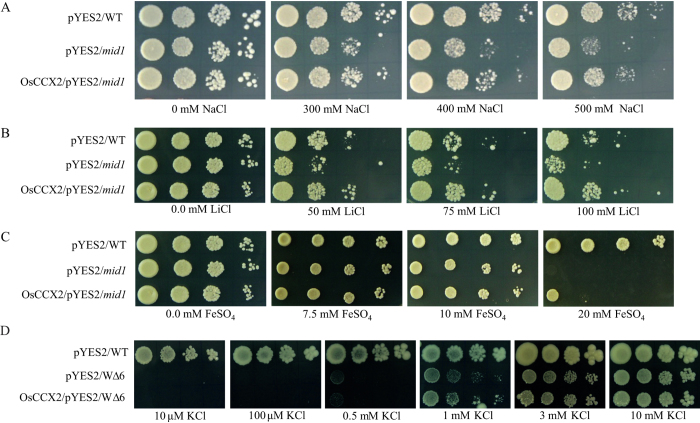
OsCCX2 provides tolerance to mid1 mutant to excess Na^+^, Li^+^ and Fe^2+^ ions while does not complement ∆*trk1*∆*trk2* mutant, wΔ6, under low K^+^ condition. (**A**) The *mid1* mutant shows sensitivity under excess (**A**) NaCl, (**B**) LiCl and (**C**) FeSO_4_. Yeast strains transformed either with empty vector or OsCCX2 were grown in SC-Ura+Gal overnight at 30 °C and diluted to 0.5 OD_600_ followed by 10 times serial dilutions of 0.5 OD_600_ were dotted on SC-URA+Gal containing different concentrations of NaCl, LiCl and FeSO_4._ The plates were incubated for 3 days. OsCCX2 was able to suppress the Na^+^, Li^+^ and Fe^2+^ sensitivity of *mid1* mutant and hence provide tolerance toward these metal ions. (**D**) The high affinity K^+^ uptake in yeast W∆6 mutant is compromised. OsCCX2 was unable to complement W∆6 mutant and showed growth like vector control under low K^+^ concentrations on solid arginine-phosphate medium as described under “Material and methods” section. The plates were incubated for 5 days.

As mentioned above, we have shown that OsCCX2 has ability to suppress Ca^2+^, Na^+^, Li^+^, Fe^2+^, Zn^2+^ and Co^2+^ sensitivity of K667 mutant. In order to further validate the metal transport properties in another yeast mutant, we choose the *S. cerevisiae mid1* mutant. The *MID1* gene is nonessential for vegetative growth. Generally, *mid1* mutant cells grow normally in a medium having glucose as a carbon source even at higher concentration of NaCl (upto 500 mM) while *mid1* mutant shows impaired growth in presence of galactose at higher concentration of NaCl (upto 500 mM). The galactose inducible pYES2 empty vector expressed in *mid1* mutant showed impaired growth at higher concentration of NaCl (500 mM), LiCl (25–100 mM) and FeSO_4_ (20 mM) in presence of galactose as carbon source and as an inducer of *Gal1* promoter (Fig. 6A–C). Therefore, OsCCX2 expressing *mid1* yeast cells can suppress Na^+^, Li^+^ and Fe^2+^ ion sensitivity and provide tolerance at increasing concentration of these metal ions (Fig. 6A–C). This complementation further validates the cation transport activity of OsCCX2 in another yeast mutant background.

### Unlike AtCCXs, OsCCX2 could not transport K^+^

The AtCCX3 and AtCCX5 shows K^+^ transport in yeast mutant. To validate K^+^ transport activity of OsCCX2, we transformed it into W∆6 yeast double mutant^43^. The W∆6 mutant lacks *Trk1* and *Trk2* genes and hence a high affinity K^+^ transport is compromised in low K^+^- Na^+^ medium. The arginine-phosphate yeast growth medium was specially designed with lesser K^+^ and Na^+^ content for evaluating the growth sensitivity of W∆6 yeast double mutant^43^. Similar to W∆6 mutant, OsCCX2 expressing W∆6 cells were not able to complement the mutant under micromolar concentration of K^+^ in arginine-phosphate medium and suggest its inability to transport K^+^ under low K^+^- Na^+^ medium (Fig. 6D).

### OsCCX2 expressing K667 cells have lesser cation content

**Figure 7:**
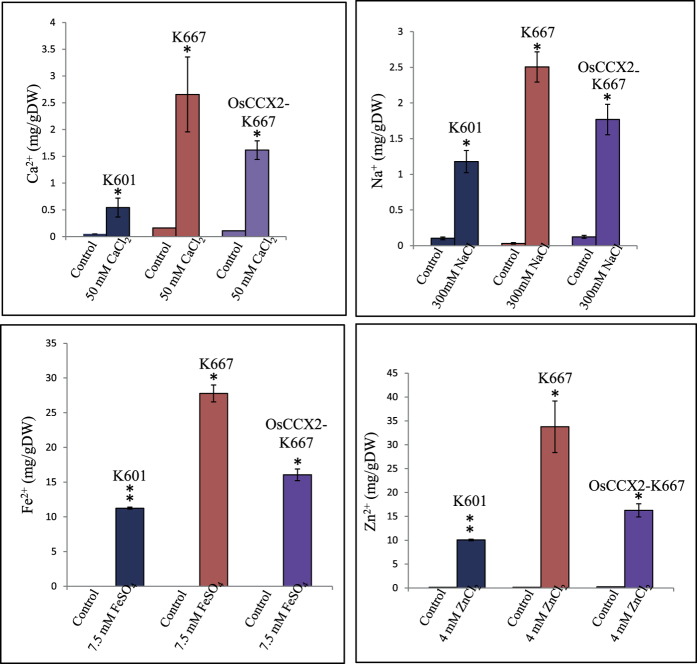
Total Ca^2+^, Na^+^, Fe^2+^ and Zn^2+^ accumulation in K667 yeast mutant expressing OsCCX2 under normal and excess metal concentrations. Yeast strains transformed either with empty vector or OsCCX2 were grown without excess metal (control) or with indicated excess metal ions. The cells were harvested and samples were prepared for atomic absorption spectrophotometric analysis as described under “Material and methods” section. The total metal ion content in yeast was measured by atomic absorption spectrophotometry. Differences between mean values of treatments and controls were compared using Student’s t -tests (^*^P≤0.05, ^**^P≤0.01).

The yeast cells maintain cationic balance by various signaling pathways. Earlier results of this study suggest that OsCCX2 suppress Ca^2+^, Na^+^, Li^+^, Fe^2+^, Zn^2+^ and Co^2+^ ion sensitivity of K667 yeast mutant (Figs 3, 4, 5). To understand the differential accumulation of these above-mentioned ions in the yeast, we have analysed the total cation content of wild type yeast (K601), K667 mutant and K667 mutant expressing OsCCX2 under excess concentration of CaCl_2_, NaCl, FeSO_4_ and ZnCl_2_ as mentioned in material and methods. Atomic absorption spectrophotometric analysis was done to estimate the total accumulation of Ca^2+^, Na^+^, Fe^2+^ and Zn^2+^ elements in yeast cells. The total contents of Ca^2+^, Na^+^, Fe^2+^ and Zn^2+^ ions under normal growth condition were comparable to standard metal contents of yeast cells^48^ (Fig. 7).

It is already known that total Ca^2+^ concentration in K601 strain (WT) is below 0.2 mg/g dry weight^49^, which corresponds well with the results of this study (Fig. 7). The total concentrations of Ca^2+^ and Na^+^ ions were in ppm range while total heavy metal Fe^2+^ and Zn^2+^ contents were in ppb range as reported previously^49^ (Fig. 7). The K667 mutant accumulates more Ca^2+^, Na^+^, Fe^2+^ and Zn^2+^ ions than WT and OsCCX2 expressing K667 cells. The wild type strain has lowest Ca^2+^, Na^+^, Fe^2+^ and Zn^2+^ content than either OsCCX2 expressing K667 cells or empty vector expressing K667 cells under excess metal concentrations (Fig. 7). These observations were statistically supported with significant p-value.

## Discussion

Cation/Ca^2+^ exchangers from lower to higher plants indicate their vital role in maintaining calcium-cation homeostasis in the plant cells. To characterize an individual gene or group of genes, expression analysis under various conditions may provide clues about their possible functions in the cell. Rice *OsCCX* genes are responsive to abiotic stresses, especially transcripts of *OsCCX2* were differentially upregulated under drought and salt stresses (Fig. 1A). Earlier reports also indicate that expression of *Arabidopsis CCX1* increases under drought and osmotic stresses while *CCX2* transcript is upregulated under cold stress^32^. *Arabidopsis CCX3* transcript is also upregulated under NaCl and KCl treatment while expression of *CCX4* does not change under abiotic stresses^32^. The expressional analysis of *OsCCXs*, especially *OsCCX2*, at various time points under abiotic stresses indicates its possible role in abiotic stress signaling in rice.

In plant cell, calcium ATPases and calcium exchangers maintain nanomolar [Ca^2+^]_cyt_ by efflux or influx of Ca^2+^ present either at plasma membrane or at endomembranes^50^,^51^. Under Ca^2+^ deficiency, the transcript level of ATPases and CAXs remains to be either unchanged or downregulated, to maintain optimal level of Ca^2+^ in cytosol. Microarray expression data under 5 days or 14 days of Ca^2+^ deficiency shows moderate decrease in expression of *OsCCX1* and *OsCCX3* while *OsCCX2* transcripts were highly down regulated (Fig 1E). Moreover, *OsCCX2* transcripts were slightly upregulated by six hours of Ca^2+^ resupply upon Ca^2+^ deficiency (Fig. 1F). Transcriptome analysis revealed that most of the *Arabidopsis* Ca^2+^ ATPases are down-regulated under Ca^2+^ deficiency^52^. It was found that under Ca^2+^ deficiency, *OsCAX1a, CAX2* transcript was significantly down regulated^40^. This role was further supported by transcriptome analysis of *Arabidopsis* Ca^2+^ exchanger, *CAX1*, which showed upregulation in response to exogenous Ca^2+^ application^53^. Therefore, altered expression of *OsCCX2* under Ca^2+^ deficiency/resupply indicate its role in Ca^2+^ homeostasis in plant cell.

Subcellular localization of OsCCX2 in *Nicotiana benthamina* epidermal peel cells revealed that it is localized to vacuolar membrane. In earlier study also, AtCCX3 was shown to be localized to vacuolar membrane of onion peel cells^32^ while AtCCX5 was localized to plasma membrane and around the nuclear periphery in yeast cells^33^. Based on these report and the results of the present study, it is speculated that different members of CCXs protein might be involved in transporting the ion across various membrane of cell. However, this assumption requires in-depth functional assays in future studies.

Yeast mutant complementation analysis has been extensively used to assess the functional nature of most of the Ca^2+^ exchangers and pumps of plants^40^,^41^,^49^,^50^. Previously, it is well established that plant CAXs have ability to suppress Ca^2+^ sensitivity of K667 yeast mutants^30^,^41^,^42^ but AtCCX3 and AtCCX5 were unable to complement and suppress Ca^2+^ sensitivity of this yeast mutant. In contrast to previously characterized CCXs, rice OsCCX2 complements the K667 yeast mutant in presence of excess Ca^2+^ like CAXs members. This result possibly infers that OsCCX2 shows similarity in Ca^2+^ transport properties like *Arabidopsis* CAXs and others. Unlike majority of plant CAXs, full length OsCCX2 was able to rescue CaCl_2_ sensitivity of K667 mutant, indicating absence of N-terminal regulatory domain.

The OsCCX2 protein complements the NaCl sensitivity of K667 mutant in presence of excess NaCl. It was reported that AtCCX5 showed Na^+^ transport in 9.3 yeast mutant cells (defective in K^+^ and Na^+^ transport) under low Na^+^-K^+^ medium^33^. It also provide tolerance to Li^+^ indicating its unique Li^+^ ion transport unlike reported for *Arabidopsis* CAXs and CCXs. As reported previously, Li^+^ could compete with Na^+^ transport in yeast cells^54^,^55^. Soybean cation/proton antiporter, *GmCAX1* expression is induced by Ca^2+^, Na^+^ and Li^+^ treatments^56^. *Arabidopsis* plants overexpressing *GmCAX1* were more tolerant to elevated Li^+^ and Na^+^ levels during germination and suggested that GmCAX1 may function as antiporter for Na^+^ and Li^+^^56^. It is quite possible that OsCCX2 might also be acting as Na^+^ and Li^+^ transporter similar to GmCAX1. Interestingly, based on the *∆trk1trk2* yeast mutant complementation analysis, OsCCX2 does not have the ability to transport K^+^ ions like reported for other AtCCXs. Therefore, OsCCX2 have Na^+^ transport activity like AtCCX5 while in contrast to AtCCX3 and AtCCX5, unable to transport K^+^ in yeast mutant cells.

OsCCX2 shows similar properties like IB type of CAXs, which also transport heavy metals such as Fe^2+^, Zn^2+^ and Co^2+^ and provide tolerance to K667 mutant. *Arabidopsis* AtCCX3 and AtCCX5 also transport divalent heavy metal ions such as Mn^2+^, Zn^2+^ and Co^2+^, Cd^2+^, Li^+^ ions respectively by unknown mechanism^32^,^33^.

In contrary to our expectation, OsCCX2 was unable to complement the single metal transport defective yeast mutants *ccc1, zrc1, cot1* (defective in Fe^2+^, Zn^2+^ and Co^2+^ transport respectively) under excess of respective metal ions (Fig. S4). In a previous report as well, *Arabidopsis* ZAT1, a putative vacuolar Zn^2+^ transporter, failed to suppress the Zn^2+^ sensitivity of *zrc1cot1* double mutant strain but it was able to suppress a *Schizosaccharomyces pombe* Zn^2+^-sensitive mutant devoid of *SpZRC1* gene^57^. It is quite possible that OsCCX2 is not able to complement these specific mutants, which are generated in *Saccharomyces cerevisiae* background. Hence, it is quite apparent that rice OsCCX2 might show functional heterogeneity (variability in transport) towards different metal ions transport and indicate its role in Fe^2+^, Zn^2+^ and Co^2+^ ions transport.

To further validate the cation transport activity in other mutants than K667, we choose *mid1* mutant for complementation analysis in this study. MID1 is N-glycosylated integral membrane Ca^2+^-permeable channel required for Ca^2+^ influx and stimulated by mating pheromone α-factor^58^. The *mid1* mutant also show impaired growth in presence of excess NaCl and metal ions when galactose is used as a carbon source. We showed that *mid1* mutant expressing OsCCX2 shows more tolerant phenotype in presence of galactose along with excess NaCl, LiCl and FeSO_4_. At present, we do not know the mechanism of tolerance provided to *mid1* mutant by OsCCX2 in above-mentioned conditions but it may be speculated that OsCCX2 might be involved in excess metal ions transport and hence impart tolerance to *mid1* mutant.

Here, we have shown that total Ca^2+^, Na^+^, Fe^2+^ and Zn^2+^ ions accumulation is higher in K667 mutant than OsCCX2 expressing K667 mutant when grown in excess of these metal ions. We hypothesize that the higher accumulation of these cations leads to sensitive phenotype of K667 mutant but this notion require further experimental evidences. It was already reported that another yeast triple mutant, K616, where high affinity Ca^2+^ uptake is compromised, accumulates 8 times higher Ca^2+^ than WT when subjected to 20 nM external Ca^2+^^49^. Moreover, deletion of CNB1 blocks ENA1 expression and hence regulates ENA1 mediated efflux of cations. Therefore, calcineurin signaling plays critical role in cation homeostasis and ENA1 mediated cation efflux^59^,^60^,^61^. OsCCX2 complements K667 mutant, which lacks *PMC1*, *VCX1* and *CNB1* genes. It is possible that in absence of functional calcineurin, expression of ENA1 system genes is compromised and therefore lesser efflux of excess cations like Na^+^, Li^+^ and other cation through ENA efflux system and hence higher accumulation of cations take place, especially Na^+^ in K667 mutant.

Overall, this study suggests that OsCCX2 is involved in providing tolerance to K667 mutant against over-accumulation of Ca^2+^, Na^+^, Fe^2+^ and Zn^2+^ in yeast cells. Hence, it leads to survival of OsCCX2 expressing K667 mutant under excess metal ions by some unknown mechanism, which requires further experimental work in near future.

### Conclusion and future prospective

**Figure 8:**
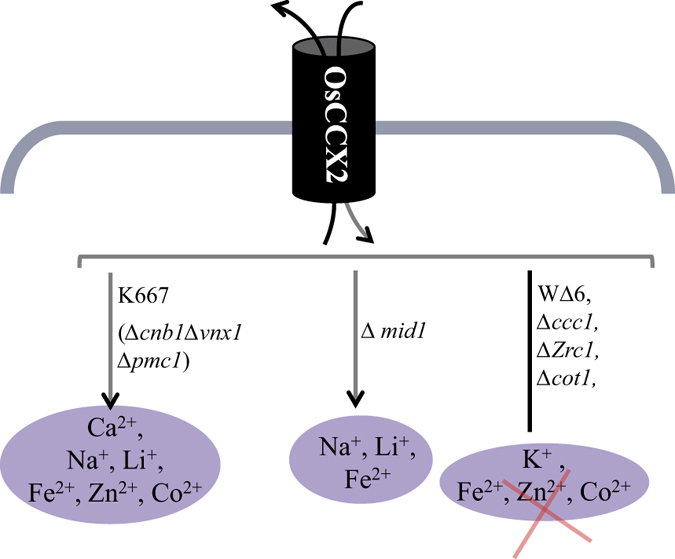
A hypothetical model depicting transport by tonoplast localized OsCCX2 of monovalent as well as divalent metal ions. OsCCX2 provide tolerance to K667 yeast mutant under excess CaCl_2,_ NaCl and heavy metals. OsCCX2 also provide tolerance to *mid1* mutant under excess NaCl and heavy metals while it does not complements K^+^ uptake deficient mutant, WΔ6, under K^+^ deficient condition. Therefore OsCCX2 mediates transport of divalent (Ca^2+^, Fe^2+^, Zn^2+^ and Co^2+^) as well as monovalent cations (Na^+^ and Li^+^) in different yeast mutants.

In conclusion, rice OsCCX2, belongs to Ca^2+^/cation exchanger family. In contrast to other rice CCXs members, *OsCCX2* transcript is highly inducible under abiotic stresses especially drought and salt and also under exogenous application of ABA. The expression of *OsCCX2* is downregulated in seedlings grown under Ca^2+^ deficient conditions and the protein is localized to vacuolar membrane of *N. benthamiana* epidermal peel cells. The bioinformatic analysis reveals that OsCCX2 is a Ca^2+^/Na^+^ exchanger, with 12 transmembrane domains and peculiar cation exchange domains. OsCCX2 suppresses the Ca^2+^, Na^+^, Li^+^, Fe^2+^, Zn^2+^ and Co^2+^ sensitivity of low affinity Ca^2+^ uptake deficient yeast K667 mutant and Na^+^, Li^+^ and Fe^2+^ sensitivity of *mid1* mutant (Fig. 8). Whereas, OsCCX2 could not suppress W∆6 (∆*trk1trk2*) yeast mutant suggesting inability to transport K^+^ ions under low K^+^ condition (Fig. 8). In conclusion, we have identified rice OsCCX2, a tonoplast localized Ca^2+^/cation exchanger showing a distinct monovalent and divalent cations exchange properties in yeast cells.

In future, *in planta* characterization of OsCCX2 under abiotic stress and different metal ions will reveal its role in cation homeostasis in rice. Genetically engineered variants of CCX2 proteins can be used as potential candidate for enhancing the abiotic stress tolerance, increased level of calcium content in edible parts of plant to eradicate Ca^2+^ malnutrition, and can be used for phytoremediation of heavy metals polluted soil.

## Materials and Methods

### Microarray based gene expression analysis of *OsCCXs*

The microarray expression profiles of all the rice *CCXs* under different abiotic stresses were retrieved from publically available database, Rice DB:*Oryza* Information Portal (http://ricedb.plantenergy.uwa.edu.au/). Transcript profiling of rice *OsCCXs* in Ca^2+^ deficient conditions was analysed from the microarray expression data of rice seedling^36^. We could not monitor the expression of *OsCCX4* because probesets for *OsCCX4* were not available in rice microarray chips (Agilent and Affimetrix).

### Expression analysis of OsCCX2 by quantitative PCR

Microarray expression data of *OsCCX2* under abiotic stress conditions and exogenous ABA application showed significant differential regulation, validated by quantitative PCR (qPCR) using two biological replicates. The microarray expression pattern of *OsCCX2* under 5 days and 14 days of Ca^2+^ deficiency and 6 hrs of resupply were also validated by qPCR. The primers of *OsCCX2* were designed from 3′ UTR region using PRIMER EXPRESS (PE Applied Biosystems, USA) with default parameters and analyzed using BLAST tool of NCBI and dissociation curve analysis after the PCR reaction for their specificity (Table S1). First strand cDNA was synthesized using 2 μg of DNaseI treated total RNA, in a 50 μl reaction volume, using high-capacity cDNA Archive kit (Applied Biosystems, USA). KAPA SYBR FAST Master Mix (KAPA BIOSYSTEMS, USA) was used to determine the expression levels of respective genes by ABI Prism 7000 Sequence detection System (Applied Biosystems, USA). Biological duplicates of each sample were accounted for expression analysis by qPCR. The average Ct values were calculated by accounting the three technical replicates for each sample. The cDNA variance among samples was normalized with expression value of *ACTIN2*, as an endogenous control. Relative expression values were calculated by ΔΔCt method and normalized the data against the maximum average expression value from microarray.

### Sub-cellular localization of OsCCX2 into *Nicotiana benthamiana* epidermal peels

Agrobacterium *tumefaciens* (GV3101: pMP90) was transformed with OsCCX2-GFP and GFP-OsCCX2 constructs. Overnight grown culture of transformed *Agrobacterium* (OD_600_ _=_ 0.5) and helper strain p19 cells (OD_600_ = 0.3) were pelleted down at 5000g for 15 minutes. Pelleted cells were re-suspended in activation buffer (10 mM MES, 10 mM MgCl_2_, 150 μM acetosyringone) and kept for 2–4 hours at room tempreture. The re-suspended cells were then used to infiltrate the leaves of 4–6 weeks old *N. benthamiana* plants. After infiltration, plants were kept for incubation (3–4 days) under 12 h light/12 h dark photoperiodic cycle at 28 °C. *Agrobacterium*-infiltrated *N. benthamiana* epidermal peel cells were analyzed in TCS SP5 laser scanning confocal microscope (Leica, Germany) for sub-cellular localization and co-localization experiments. GFP fluorescence signals were detected at 500 to 535 nm after excitation at 488 nm while mCherry was excited at 543 nm and scanned at 600–630 nm. For co-localization experiments, sequential scanning was done for both the channels and then merged together to shows overlapping signals. All the images were further processed using Leica LAS AF Lite software.

### DNA manipulation- Cloning of *OsCCX2* and plasmid DNA constructs

Rice *OsCCX2* was amplified from cDNA of 7 day old IR-64 rice seedlings and was cloned into pJET1.2 vector (Fermentas Inc. USA). The cloned gene was confirmed by sequencing. *OsCCX2* was subcloned into galactose inducible yeast expression vector, pYES2 (Clontech Inc. USA). For *in planta* subcellular localization, *OsCCX2* ORF was cloned into pGPTVII-GFP.Kan vector to form fusion protein at the N-terminal of GFP (OsCCX2-GFP)^62^. To generate the fusion protein at C-terminal of GFP (GFP-OsCCX2P), OsCCX2 entry clone was mobilized to pSITE2CA vector^63^. Both the resulting constructs contained cauliflower mosaic virus (CaMV) 35S promoter driving expression of the transporter. All primers used in this study are listed in Table S1.

### Yeast strains, media and growth conditions

To test the different cations transport function of OsCCX2 in yeast, OsCCX2/pYES2 along with vector control, were individually transformed into different yeast mutant strains by LiAc/ss carrier DNA/PEG method^64^. The details of different *Saccharomyces cerevisiae* yeast strains used in this study are listed in Table S2. Transformants were selected for uracil prototrophy by plating on synthetic medium minus uracil (SC-Ura; 6.7 g/L yeast nitrogen base without ammonium sulphate, without amino acids, 5 g/L ammonium sulphate, 1.92 g/L of drop-out mix without uracil, 2% glucose and 2% agar). Single colony of each transformant was grown in SC-Uracil having midlog phase and culture was pelleted, washed thrice with water. The yeast cells were serially diluted with water to obtain 10^−1^, 10^−2^ and 10^−3^ fold dilutions of 0.5 OD_600_ cultures. 5 μl of each serial dilution was dotted on the SC-Uracil/galactose media with mentioned different concentrations of cations. To observe the growth, plates were incubated at 30 °C for 3 days otherwise mentioned in figures.

Arginine-phosphate (AP) medium, first described by Rodriguez-Navarro *et al*. (1984) is a Na^+^- K^+^ -free minimal synthetic medium frequently used to examine the effect of Na^+^ and K^+^ on *∆trk1∆trk2* mutant growth^43^. The composition of AP medium: 8 mM phosphoric acid, 10 mM L-arginine, 2.0 mM MgSO_4_, 0.2 mM CaCl_2_, 10 mM MES, 2% glucose, recommended vitamins, trace elements, amino acids and pH 6.5 was adjusted using L- arginine. 1% agarose was used to make solid AP medium plates.

### Total Ca^2+^, Na^+^, Fe^2+^ and Zn^2+^ analysis in yeast cells

Total Ca^2+^, Na^+^, Fe^2+^ and Zn^2+^ were measured using Shimadzu Atomic Absorption Spectrophotometer 6300. Single colony of K601 having pYES2 vector and K667 mutant harbouring pYES2 and OsCCX2/pYES2 cultured in SC-uracil having different concentrations of metal ions (CaCl_2_, 50 mM; NaCl, 300 mM; FeSO_4_, 7.5 mM and ZnCl_2_, 4 mM) at a cell density of ~2 OD_600_/ml. The cultures of each set were harvested in pre- weighed microfuge tubes by centrifugation. The cells grown in CaCl_2_ were washed three times with 10 mM EGTA pH 5.5, while cells grown in other metal ions were washed with 10 mM EDTA and then finally with Milli Q water. After aspirating the supernatant, pelleted cells were dried in oven overnight at 60 °C. The dried samples were weighed again and re-suspended in HNO_3_ for atomic absorption spectrophotometry.

## Additional Information

**How to cite this article**: Yadav, A. K. *et al.* A rice tonoplastic calcium exchanger, OsCCX2 mediates Ca^2+^/cation transport in yeast. *Sci. Rep.* **5**, 17117; doi: 10.1038/srep17117 (2015).

## Supplementary Material

Supplementary Information
