# Supplementary material for: A rice tonoplastic calcium exchanger, OsCCX2 mediates Ca2+/cation transport in yeast
Source: Sci Rep. 2015 Nov 26;5:17117. doi: 10.1038/srep17117 (PMC4660821; doi:10.1038/srep17117)
Supplement: Supplementary Information [file srep17117-s1.pdf]

A rice tonoplastic calcium exchanger, OsCCX2 mediates Ca^{2+}/cation transport in yeast

Akhilesh K. Yadav, Alka Shankar, Saroj K. Jha, Poonam Kanwar, Amita Pandey, Girdhar K. Pandey*

Department of Plant Molecular Biology, University of Delhi South Campus, Benito Juarez Road, Dhaula Kuan, New Delhi-110021, India.

*Author for correspondence: Tel. +91-11-24116615, Fax. +91-11-24115270

Email: gkpandey@south.du.ac.in

Fig. S1a.

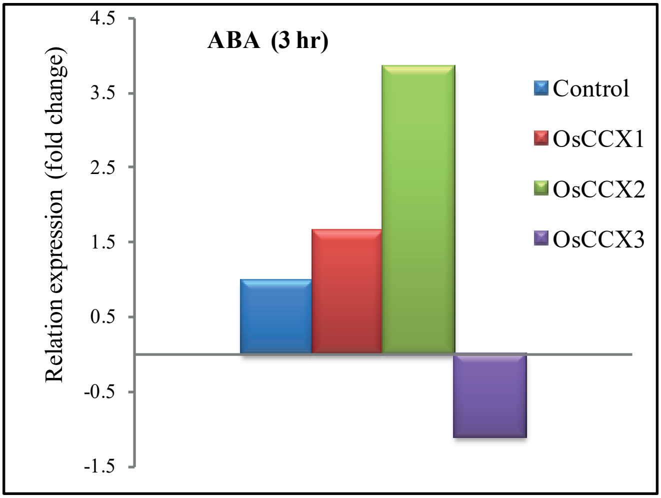

Fig. S1b.

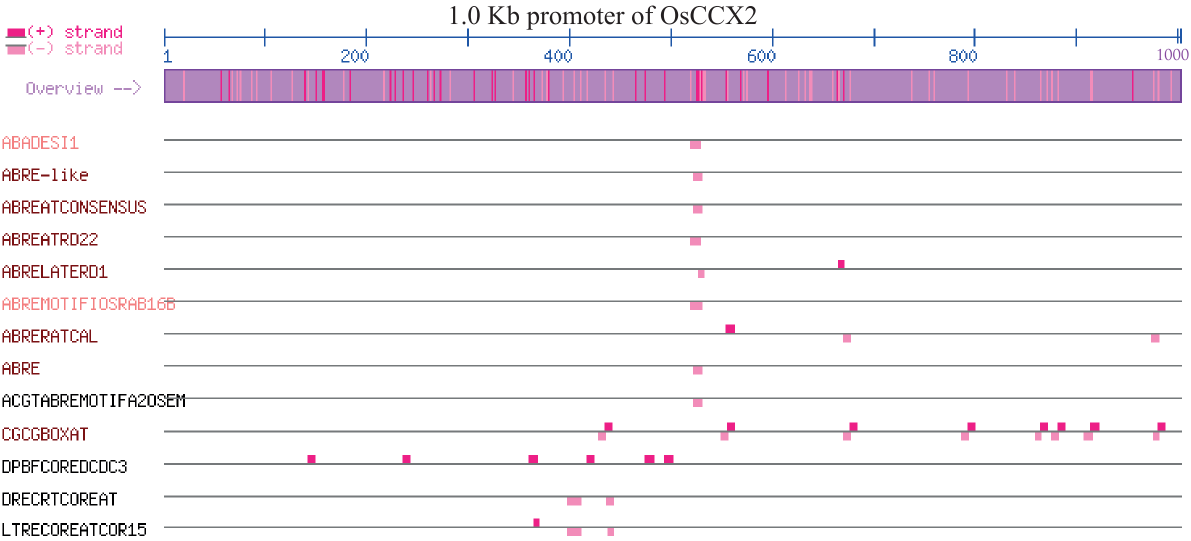

Figure S1a. *Microarray expression profile of OsCCX genes under exogenous ABA*. The rice seedling treated with ABA for 3 hrs showed upregulation of OsCCX1 and OsCCX2 while OsCCX3 is downregulated with respect to control. Y-axis depicts the relative expression value in terms of fold change. **b.** OsCCX2 promoter (1kb upstream of transcription start site) analysis shows for presence of various cis-elements. The 1000 bp upstream promoter region of OsCCX2 contains various ABA, drought, cold and calcium responsive cis-elements. The promoter analysis was carried out by plant promoter analysis navigator, PLANTPAN tool (http://plantpan.mbc.nctu.edu.tw/seq_analysis.php).

Fig. S2.

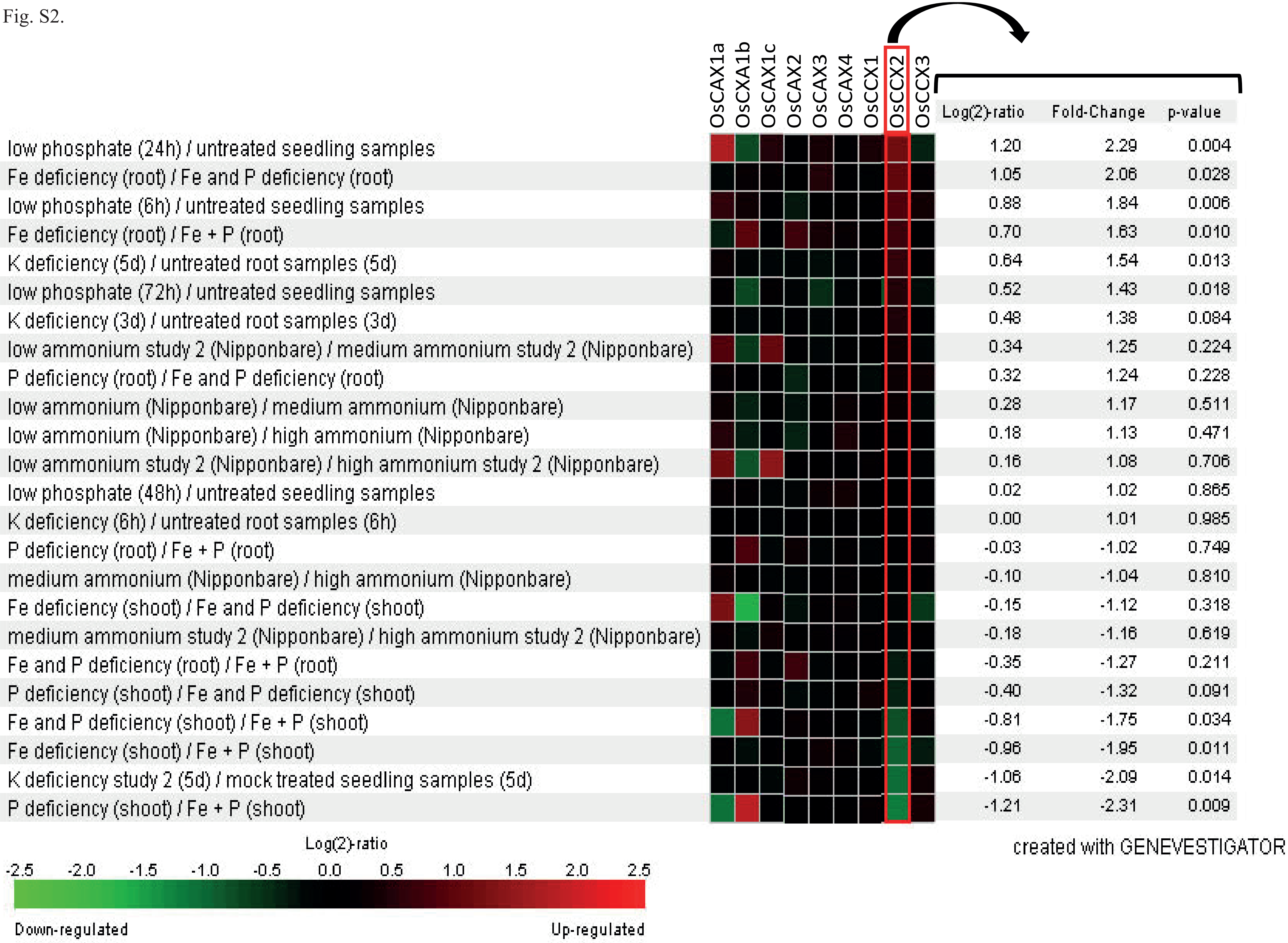

Figure S2. *Expression analysis of Rice CAXs and CCXs under various nutrient deficient conditions.* OsCCX2 was upregulated under phosphate starvation in seedling stage while shows down regulation in shoot. OsCCX2 is also down regulation under potassium starvation condition. Other CCXs were not much differentially expressed under different nutrient deficient conditions. The highlighted box indicates the expression of OsCCX2 whereas their expression and p-value are shown at the right side of heatmap. At bottom, gradient color scale bar showing green color for downregulation while red color for upregulation of genes under mentioned nutritional conditions.

Fig. S3.

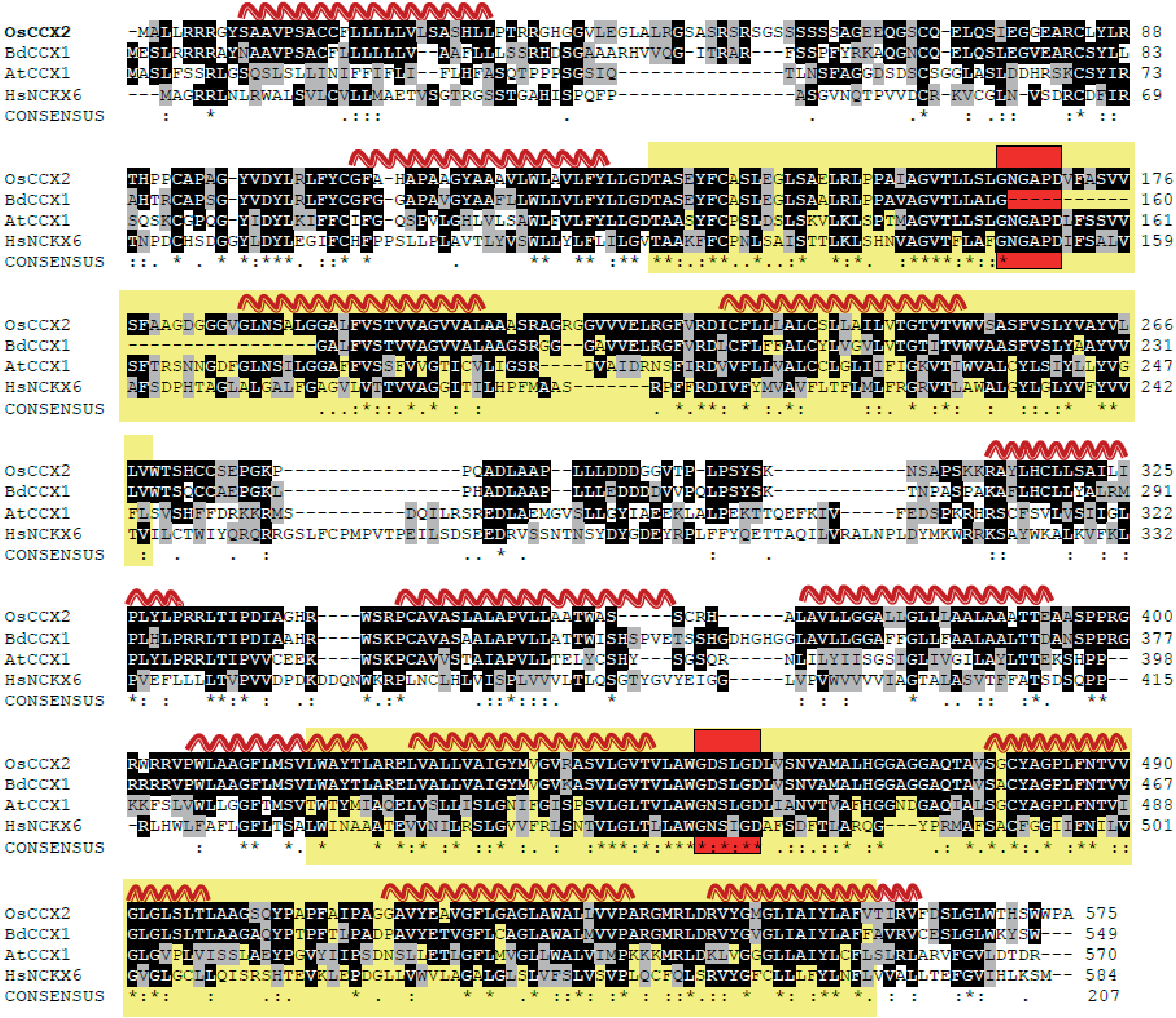

Figure S3. *Multiple sequence alignment of OsCCX2 with orthologous proteins depicting presence of conserved domains and motifs.* Protein sequences of OsCCX2 and its orthologues in Arabidopsis (AtCCX1), Brachipodium (BdCCX1), human (NCKX6) were aligned using clustalX 2.1. The 12 transmembrane domains of OsCCX2 were represented with red helixes on the top of sequence. Two Ca^{2+}-Na^{+} exchanger domains are shown with filled yellow box and characteristic α1 and α2 motifs, present in Ca^{2+}-Na^{+} exchanger domains, were marked with red box.

Fig. S4.

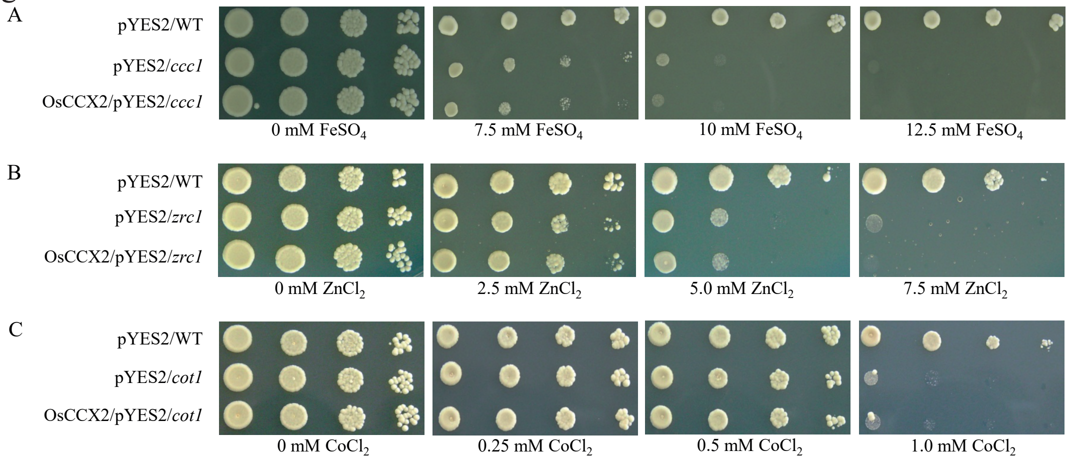

Figure S4. *OsCCX2 does not complement single mutants defective in specific metal transport.* The yeast *ccc1* (defective in Fe^{2+} transport, A), *zrc1* (defective in Zn^{2+} transport, B) and *cot1* (defective in Co^{2+} transport, C) single mutants were transformed with empty vectors and OsCCX2. Transformed yeast strains were grown in SC-Ura media overnight at 30ºC and diluted to 0.5 OD600 and 10 times serial dilutions of 0.5 OD600 were dotted on SC-URA+Gal media with mentioned metals ions. The plates were incubated at 30 ^{0}C for 4 days. All single mutant shows sensitivity towards respective excess metals ions and OsCCX2 was unable to complement them.

Table S1. List of different OsCCX2 primers used in this study

Used for	Primer name	Primer sequence (5'-3')
qPCR	OsCCCX2F	TGCACGATGTTCAGTAAGTTGCT
	OsCCCX2R	ATCTTGCACCTCGTGAACCAT
Sub- cellular localization	OsCCX2-GFPK-XbaIF'	AATCTAGAATGGCGCTCCTGCGCAG
	OsCCX2-GFPK-BamHIR'	AAGGATCCTGCTGGCCACCAGGAGT
For pENTR/D-TOPO	OsCCX2-TOPOF	CACCATGGCGCTCCTGCGCAGG
	OsCCX2-Topo R	CTATGCTGGCCACCAGGAGTG
Yeast complementation	OSCCX2 BamHI F' pYES2	AGGATCCATGGCGCTCCTGCGCAGG
	OSCCX2 EcoRI R' pYES2	CCGAATTCCTATGCTGGCCACCAGGAGTG

Table S2. Yeast strains used in this study

S. No.	Strain name	ORF mutated in yeast/mutant	Strain name/genotype
1	WT	-	K601; W303–derivative
2	K667	*VCX1, CNB1, PMC1*	K667; vcx1/Δ, cnb1:: LEU2, pmc1::TRP1
3	K616	*PMR1, CNB1, PMC1*	K616; pmr1::HIS3, cnb1::LEU2, pmc1::TRP1
4	WT	-	W303; ade2-1 can1-100 his3-11, 15 leu2-3, 112 trp1-1 ura3-1
5	WΔ6	*TRK1, TRK2*	WΔ6; Mat a ade2 ura3 trp1 trk1Δ::LEU2 trk2Δ::HIS3
6	WT	-	BY4741; MATa; his3Δ 1; leu2Δ 0; met15Δ 0; ura3Δ 0
7	*mid1*	*MID1/YNL291c*	BY4741; Mat a; his3Δ1; leu2Δ0; met15Δ0; ura3Δ0; YNL291c::kanMX4
8	*zrc1*	*ZRC1/YMR243c*	BY4741; Mat a; his3Δ1; leu2Δ0; met15Δ0; ura3Δ0; YMR243c::kanMX4
9	*ccc1*	*CCC1/YLR220w*	BY4741; Mat a; his3Δ1; leu2Δ0; met15Δ0; ura3Δ0; YLR220w::kanMX4
10	*cot1*	*COT1/YOR316c*	BY4741; Mat a; his3Δ1; leu2Δ0; met15Δ0; ura3Δ0; YOR316c::kanMX4
